# Hearing Aid Use and Associated Factors in South Korea

**DOI:** 10.1097/MD.0000000000001580

**Published:** 2015-10-23

**Authors:** Il Joon Moon, Sun Young Baek, Yang-Sun Cho

**Affiliations:** From the Department of Otorhinolaryngology—Head and Neck Surgery, Samsung Medical Center, Sungkyunkwan University School of Medicine, Seoul, South Korea (IJM, Y-SC); and Biostatistics team, and Samsung Biomedical Research Institute, Seoul, South Korea (SYB).

## Abstract

Despite the high prevalence of hearing impairment in the elderly, the rate of hearing aid use is still low. The objectives of this study were to report the nation-wide prevalence of hearing aid use in the Korean population and to determine the associated factors with hearing aid use utilizing a nationally representative data set.

We obtained data from the 2010 to 2012 Korea National Health and Nutrition Examination Surveys, which were cross-sectional surveys of the civilian, noninstitutionalized population of the Republic of Korea at age ≥40 years (N = 12,709). A field survey team performed interviews as well as physical examinations. Hearing aid use was assessed using an interviewer-administered questionnaire and pure-tone audiometry was administered for all participants in a sound-attenuating booth. Prevalence of hearing aid acquisition and regular use were calculated in participants who reported perceived hearing loss and who have bilateral hearing thresholds exceeding the 40 dB hearing level. Multivariable analyses were used to examine the associated factors with hearing aid use.

The prevalence of hearing aid acquisition and regular use was 17.4% and 12.6%, respectively, in South Korea. Increased hearing threshold (OR 1.05, 95% CI 1.03–1.07), severe perceived hearing loss (OR 10.73, 95% CI 4.52–25.46), annoying tinnitus (OR 3.30, 95% CI 1.61–6.74), balance problems (OR 0.39, 95% CI 0.18–0.86), and myopia (OR 0.30, 95% CI 0.12–0.76) were associated factors of regular use of hearing aids.

The prevalence of hearing aid use in Korea is relatively low. Finding relevant factors of hearing aid use could provide further insight in setting up hearing-rehabilitation strategy for the elderly with significant hearing loss.

## INTRODUCTION

Hearing loss is a common sensory impairment in the elderly, with consequences on cognitive, emotional, and social function, and prevalence of hearing loss dramatically increases with age.^1,2^ In South Korea, overall prevalence of subjective hearing loss was 12.0%, and 44.7% of the elderly (> 70 years old) reported hearing loss.^3^ As the global population increasingly ages, prevalence of hearing loss will continue to rise. Although hearing loss is highly prevalent in elderly adults and most can be successfully rehabilitated by using appropriate hearing aids, the uptake and use of hearing aids is still poor.^4^ Several studies have reported that a large proportion of people who could benefit from hearing aids do not wear them.^5,6^ The reported prevalence of hearing aid use among those with a hearing loss is low, ranging from 14.2% to 33.1% (Table 1).^7–9^ Moreover, many people provided with hearing aids are not satisfied with them and do not use regularly.^5^ Therefore, it is important to identify the associated factors with hearing aid use and identify the principal causes of nonadoption.

**TABLE 1:**
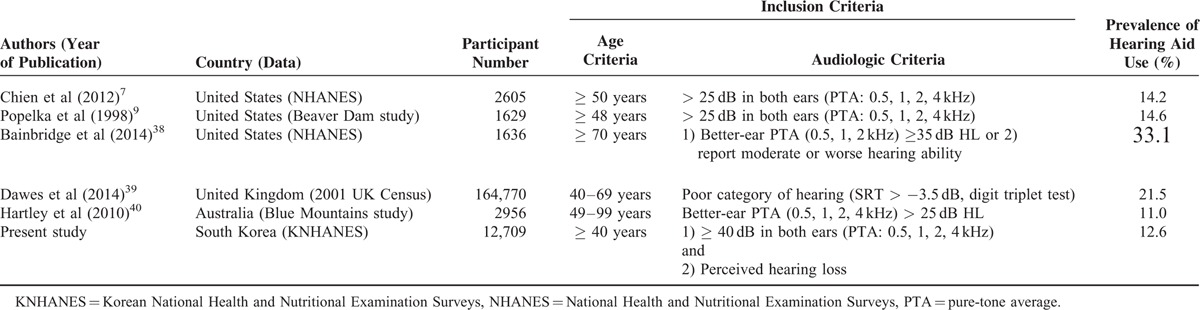
Prevalence of Hearing Aid Use in Previous Reports and Present Study

Nation-wide epidemiologic studies that are conducted by government organizations can provide powerful data for investigating the national prevalence of specific disease and health behavior of people. The Korean National Health and Nutrition Examination Survey (KNHANES) was started in 1998 to examine the general health and nutrition status of population in South Korea. From 2010 to 2012, 10,000 to 12,000 individuals were selected annually and interviewed concerning their general health and nutrition, as well as undergoing a basic health assessment. In the otolaryngologic interview and examination, histories of tinnitus, hearing loss, and noise exposure were surveyed using structured questionnaires and endoscopic evaluations of the tympanic membrane as well as objective evaluation of hearing and balance were conducted in participants of appropriate ages.

The present study was undertaken to determine the national prevalence of hearing aid use among participants >40 years of age with perceived hearing loss in South Korea based on survey data obtained from the 2010 to 2012 KNHANES and to investigate factors associated with hearing aid use.

## METHODS

### Study Population and Data Collection

KNHANES is a national cross-sectional survey of the civilian noninstitutionalized population of South Korea. Korean Society of Otorhinolaryngology–Head and Neck Surgery and Korean Ophthalmological Society support the survey. A field survey team that included an otolaryngologist, an ophthalmologist, and nurse examiners for health assessment moved with a mobile examination unit and performed interviews, physical examinations, and laboratory testing, including blood and urine collection, a pulmonary function test, a dental examination, an ophthalmologic examination, and an otolaryngologic examination. Every year, 7000 to 10,000 individuals in ∼3500 households are selected from a panel to represent the population by using a multistage clustered and a stratified random sampling method that is based on the National Census Data. The 500 survey areas were drawn from a census of population and housing by considering the proportion of each subgroup. The participation rate of selected households in the past several cycles of KNHANES has been high, ∼80%. A total of 23,621 individuals, representing the 47,761,045 individuals in South Korea, participated in the survey from 2010 to 2012, and 12,709 individuals >40 years of age were initially included in this study. Written informed consent was obtained from all participants before the survey, and approval for this research was obtained from the Institutional Review Board of the Samsung Medical Center (IRB No. 2013-02-031).

### Hearing Survey and Otologic Examination

Participants were first asked about their perceived hearing loss. In detail, the participants were asked to rate their difficulty in hearing (“Which among the below best describes your [unaided] hearing?”) by identifying their hearing as having no difficulty, mild difficulty, significant difficulty, or as no hearing. The respondents who professed any degree of hearing difficulty were then asked about their use of hearing aids. Responses to the question “Do you use hearing aid(s)?” were “yes”, “yes, but rarely use it”, or “no”. The participants were considered to regularly use hearing aids only if they answered “yes”. To determine the prevalence of tympanic membrane perforation and cholesteatomatous conditions, including retraction pocket and otitis media with effusion, an ear examination was conducted with a 4-mm 0°-angled rigid endoscope attached to a CCD camera for all participants. The pure tone air-conduction threshold was measured in a sound-proof booth using a model GSI SA-203 automatic audiometer (Entomed Diagnostics AB, Lena Nodin, Sweden). Moderate-to-profound hearing loss was defined as > 40 dB hearing level with average air-conduction hearing thresholds measured at 0.5, 1, 2, and 3 kHz according to the recommendation from the American Academy of Otolaryngology—Head and Neck Surgery.^10^ To evaluate the experience of balance problem, participants were asked whether they had experienced dizziness or imbalance, positional dizziness, and falls in the absence of external forces in the past 12 months.

### Statistical Analyses

The prevalence and 95% confidence interval (CI) for hearing aid use were calculated. To examine the association of various covariates on the use of hearing aids, univariable analysis and multivariable analysis were performed using the logistic regression model. Biologically and medically plausible covariates, as well as the variables found to be possibly associated (*P* < 0.20) in the univariable analysis were entered in the multivariable analysis model. To reflect national population estimates, sample weights were applied in all analyses. *P* values and 95% CI were corrected by Bonferroni's method in the case of multiple testing. In all tests, *P* values were 2 sided, and *P* < 0.05 was considered to be statistically significant. Statistical analysis was performed using SAS 9.3 (SAS Institute Inc, Cary, NC).

## RESULTS

### Prevalence of Hearing Aid Use in Bilateral Moderate Hearing Loss

Among the 12,709 participants, 993 had bilateral moderate to profound sensorineural hearing loss (PTA > 40 dB in both ears). Among these, those who did not respond to the questionnaire queries of hearing aid use, tinnitus, noise exposure, and perceived hearing loss, or those who did not underwent otolaryngologic or ophthalmologic examinations were excluded. Additionally, those who had no perceived hearing loss (who responded to have “no difficulty” in subjective hearing) were also excluded. Finally, 530 participants, representing 732,618 individuals with objective bilateral moderate-to-profound hearing loss (≥ 40 dB) and perceived they had hearing loss, were included in this study. Among the 530 participants, 94 (17.4%, weighted frequency 127,368) answered that they had hearing aids. However, 25 participants (4.8%, weighted frequency 35,362) rarely used hearing aids even though they had them. Thus, the remaining 69 participants (weighted frequency 92,276) who regularly used hearing aids in daily life were considered as hearing-aid user; the prevalence of hearing aid use was 12.6% (95% CI 9.0%–16.2%).

### Factors Associated with Hearing Aid Use

Hearing aid use in participants >40 years of age with bilateral moderate-to-profound hearing loss and associated factors were investigated using univariable and multivariable analyses. Table 2 displays the results of univariable and multivariable logistic analyses. In univariable analysis, hearing threshold (OR 1.06, 95% CI 1.03–1.08), perceived hearing loss (OR 13.18, 95% CI 9.07–28.65), annoying tinnitus (OR 2.61, 95% CI 1.38–4.93), tympanic membrane perforation (OR 3.22, 95% CI 1.53–6.77), myopia (OR 0.31, 95% CI 0.14–0.72), and astigmatism (OR 0.40, 95% CI 0.18–0.88) were significantly associated with hearing aid use. Multivariable logistic regression analyses were performed to evaluate relevant associated factors with hearing aid use. Of the 9 variables entered into the multivariable model, high hearing threshold (OR 1.05, 95% CI 1.03–1.07), severe perceived hearing loss (significant hearing loss or no hearing) (OR 10.73, 95% CI 4.52–25.46), annoying tinnitus (OR 3.30, 95% CI 1.61–6.74), balance problems (OR 0.39, 95% CI 0.18–0.86), and myopia (OR 0.30, 95% CI 0.12–0.76) remained as associated factors of hearing aid use.

**TABLE 2:**
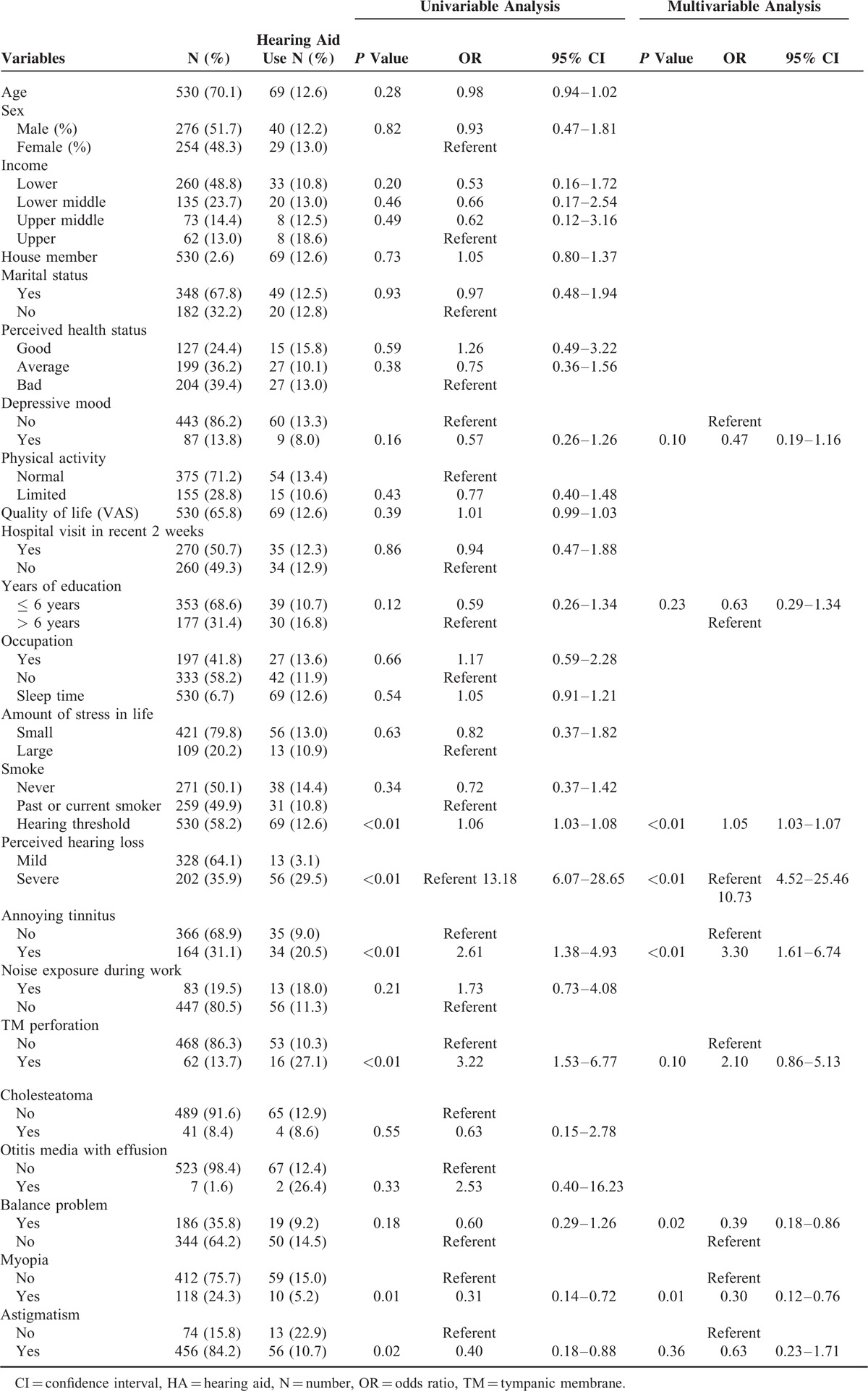
Univariable and Multivariable Analyses of Factors Potentially Associated with Hearing Aid Use in Participants > 40 Years Old with Bilateral Moderate-to-Profound Hearing Loss

### Factors Associated with Regular Hearing Aid Use

Among 94 participants who had hearing aids, only 69 participants regularly used their hearing aids and the remaining 25 participants were nonusers. Thus, in order to identify factors associated with regular hearing aid use in all hearing aid owners, additional univariable and multivariable analyses were performed. Table 3 displays the results of univariable and multivariable logistic analyses. Variables found to be possibly associated (*P* < 0.20) in the univariable analysis were entered in the multivariable analysis model. Multivariable logistic regression analysis revealed that high hearing threshold (OR 1.06, 95% CI 1.01–1.11) and number of house members (OR 1.65, 95% CI 1.12–2.44) were significant associated factors of regular hearing aid use among hearing aid owners.

**TABLE 3:**
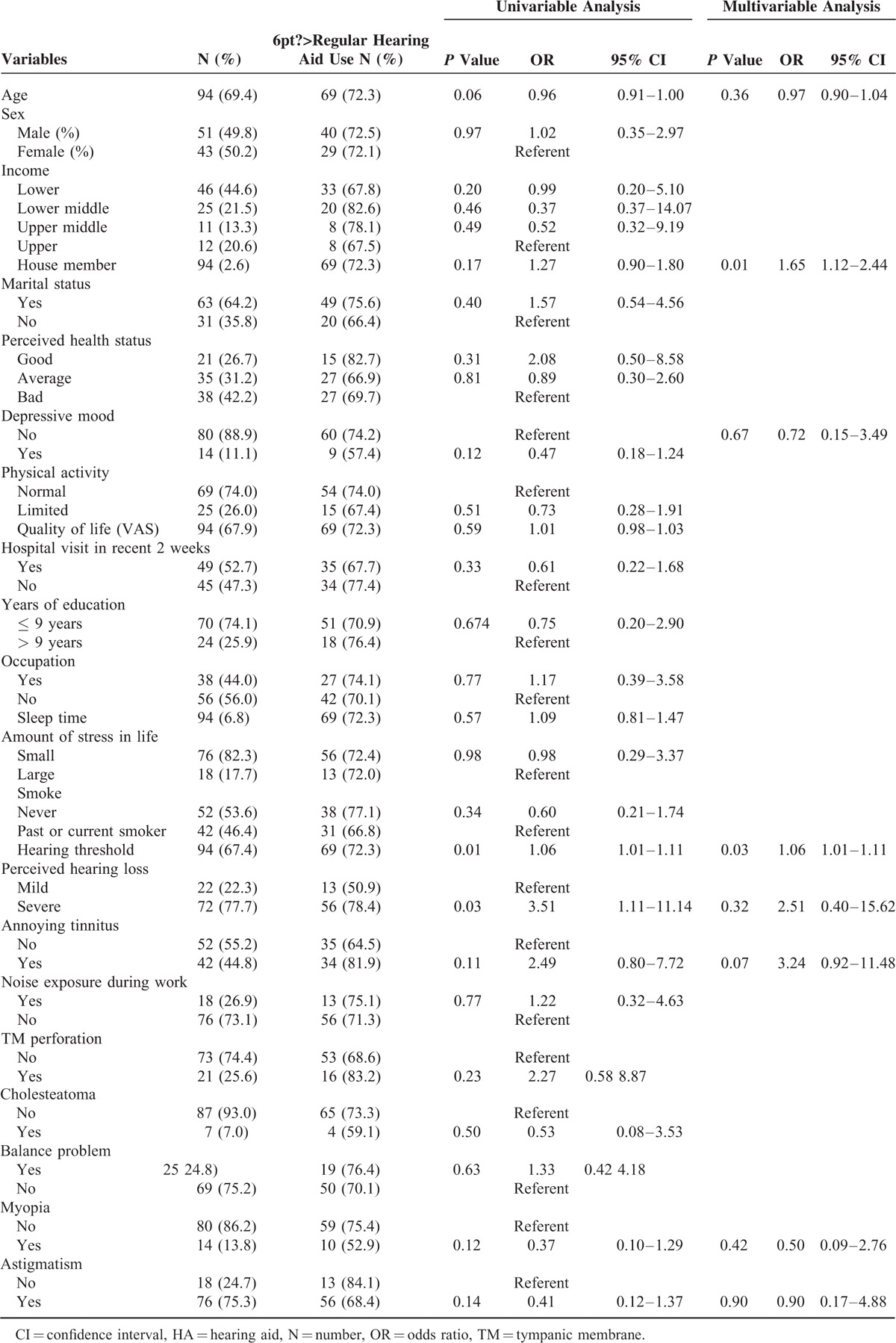
Univariable and Multivariable Analyses of Factors Potentially Associated With Regular Hearing Aid Use in Participants >40 Years Old With Bilateral Moderate-to-Profound Hearing Loss

## DISCUSSION

These are the first national estimates of hearing aid prevalence in the Korean population based on audiometric data and a large, government-centered, well-selected representative sample. Age-related hearing impairment is a hallmark of aging; therefore, the prevalence and severity increase with age.^11^ Besides its high incidence and prevalence, age-related hearing impairment affects quality of life enormously by reducing communicative relationships, as well as reducing social and emotional interactions.^12^ Thus, appropriate tools for hearing rehabilitation are needed, and hearing aids are recommended for majority of people with age-related hearing impairment. Previous studies have reported that hearing aids can improve the ability to communicate, improve quality of life, prevent social isolation, and positively affect hearing-related depression.^13–16^ In addition, by modeling different degrees of hearing loss with a multistate model, hearing aid use was proven to be a cost-effective strategy to rehabilitate the hearing-impaired elderly.^17^ However, many people with impaired hearing do not wear them. Over the last 20 years, hearing aid adoption has remained at ∼ 20% with an admitted hearing loss.^8^

In this study, national prevalence of hearing aid use in South Korea was 12.6% among people with moderate-to-profound hearing loss, which is relatively low as compared with those reported in other countries (Table 1). The estimated number of Korean adults (> 40 years of age) with moderate to profound hearing loss who do not regularly use hearing aids is 640,000. The causes for low proportion of hearing aid use among the hearing-impaired people may be related with the expenses of purchasing and maintenance, stigma about their use, negative beliefs about hearing aids, improper information or guidance about wearing them, or lack of perceived hearing handicap.^18–20^ In European countries, such as the United Kingdom, France, Denmark, and the Netherlands, expenses of hearing aids are covered by public health insurance. In the United States, the use of hearing aids has continually increased and is now ∼23%, with most of this growth attributed to free hearing aids obtained through the Department of Veterans Affairs or low-cost Internet sales.^8^ However, in South Korea, the expense of hearing aids is not covered by the government health insurance system, and the average price of hearing aids is relatively high when considering the national income per capita. In addition to cost, cultural attitudes that undervalue the role of hearing on life as well as stigma associated with hearing aid use partly account for the low rate of hearing aid use in South Korea.^21^

At present, both objective hearing thresholds and subjective assessment of participant's own hearing loss were associated with hearing aid use. People who use hearing aids have more severe hearing losses than nonusers. Considering the odds ratio of both objective and subjective hearing loss (1.05 vs 10.73), self-reported auditory difficulty is more important in using hearing aids than objective hearing sensitivity. Perceived hearing handicap assessed by the Hearing Handicap Inventory for the Elderly questionnaire is associated with the hearing aid use, with an adjusted odds ratio for a 1-unit increase in HHIE scores of 1.08.^19^ In addition, acceptance of hearing loss before hearing aid fitting positively influences both hearing aid uptake and hearing aid use.^4^ Previous studies also investigated the influence of hearing threshold level on hearing aid uptake, use, and overall satisfaction with the device. People with greater hearing thresholds reported a greater amount of hearing aid use per day and greater satisfaction levels.^22^ In another study, people who used hearing aids had a significantly higher degree of hearing loss (mean; 60 dB at 1, 2, 4 kHz) than those who declined to use the aids (mean; 48 dB).^23^

Patients with bothersome tinnitus had an increased tendency to use hearing aids in this study. Hearing aids are often recommended as a treatment option for patients who suffer from bothersome tinnitus and who have moderate to severe hearing loss. This practice is supported by recently published clinical practice guideline of tinnitus, recommending a hearing aid evaluation for patients who have persistent, bothersome tinnitus associated with documented hearing loss.^24^ The use of hearing aids in tinnitus makes the patient less aware of the tinnitus and reduces the annoying sensation that sounds and voices are masked by the tinnitus, by amplifying external sounds and subsequent activation of the auditory nervous system.^25,26^ Survey and case control studies have shown that tinnitus patients gain relief from the symptoms by the consistent use of hearing aids.^27,28^ In addition, a broader range of patients with milder degrees of hearing loss can become successful hearing aid users due to the increased use of open-fit hearing aid. This can benefit tinnitus sufferers who previously were not interested in amplification.

Presbycusis is associated with an increased risk of falls.^29^ Recently, it was shown that hearing aids are a novel treatment modality for imbalance in older adults with hearing loss.^30^ That is partly attributed to importance of auditory input in maintaining balance, because audition may represent as an independent sensory input providing spatial sound cues. However, as reported in this study, the rate of hearing aid use among people with balance problem is low, probably because of difficulties in purchasing and managing hearing aids due to restricted activity or inadequate awareness about the beneficial effect of hearing aids on balance. Balance deficits are a well-known risk factor for falls in the elderly, and both balance deficits and subsequent fear of falling are associated with restricted physical activity.^31^ Therefore, raising public awareness about the benefits of hearing aid use and appropriate rehabilitation programs to increase physical activity would be helpful in this specific population who both has hearing impairment and balance problems.

Having myopia was associated with decreased use rate of hearing aids in this study. Vision and hearing impairments are common among the elderly and can occur separately or in combination.^32^ A loss of visual ability can make it difficult for the elderly to insert a hearing aid into the ear canal, replace a battery, or provide hearing aid maintenance^33,34^ because poor vision can affect manual dexterity in aged population. Furthermore, many participants with both hearing and vision impairments express concerns about managing hearing aids due to their low vision,^35^ which may explain the low rate of hearing aid use in individuals with myopia. In addition, people with low vision often depend on spatial hearing and echolocation. However, if hearing loss is combined, then safe mobility may be compromised by loss of accuracy of sound localization and reduced perception of cues.^36^ Thus, active provision of hearing-rehabilitation services is required for this dual sensory impairment.

In this study, among 94 participants who purchased hearing aids, 25 participants (26.6%) rarely used hearing aids. This result is in line with prior research from UK, indicating that a total of 29% of hearing aid owners do not use their hearing aids on a regular basis.^37^ It is also important to identify comprehensive list of risk-factors for compliance with hearing aid use. The most important factors for regular hearing aid use (good compliance) in this study were the number of house member and hearing threshold. This result suggests that needs for communication with house (family) members can positively affect motivation of hearing-impaired listeners to use hearing aids regularly. It also indicates that hearing-impaired listeners with small family can be at higher risk for nonregular hearing aid use, and clinicians should pay attention to this specific population during the counseling and fitting of their aids.

A possible limitation of this study is the lack of comprehensive inclusion of possible associated factors that include source of motivation, attitudes toward hearing aid, expectations of hearing aids, and individual experiences of hearing-aid counseling. However, the key attributes of this study lie in the national cross-sectional survey with representative population based on the National Census Data, providing detailed and standardized audiometric data, as well as in the comprehensive range of demographic and clinical variables accurately measured by an otolaryngologist, ophthalmologist, and nurse examiners. These data also enable a thorough evaluation of associated factors with hearing aid use among older (≥40 years of age) adults in South Korea. The results could be used in devising strategies to counsel the hearing-impaired people and to increase hearing aid adoption rates.

## CONCLUSIONS

The prevalence of hearing aid use is estimated as 12.6% in South Korea. Increased hearing threshold, severe perceived hearing loss, accompanying annoying tinnitus, balance problems, and myopia are associated with hearing aid use. Among these factors, increased hearing threshold, severe perceived hearing loss, and accompanying annoying tinnitus may be easily expectable, but balance problems and myopia were newly found to be associated with hearing aid use. Therefore, concerns about balance disorder and myopia may need to be raised during hearing aid counseling. Additionally, hearing-impaired listeners with higher hearing thresholds and small family are at risk for nonregular hearing aid use.

This information should be incorporated into daily clinical practice, and interventions to reduce barriers of hearing aid use seem to be necessary in satisfying an unmet need, and to reduce negative consequences from hearing impairment in the elderly. Further researches are needed to evaluate the effect of active/professional counseling and long-term follow-up program on regular hearing aid use.

## References

[1] Huang Q Tang J Age-related hearing loss or presbycusis. *Eur Arch Otorhinolaryngol* 2010; 267:1179–1191. 20464410 10.1007/s00405-010-1270-7

[2] Lin FR Niparko JK Ferrucci L Hearing loss prevalence in the United States. *Arch Intern Med* 2011; 171:1851–1852. 22083573 10.1001/archinternmed.2011.506 PMC3564588

[3] Cho YS Choi SH Park KH Prevalence of otolaryngologic diseases in South Korea: data from the Korea national health and nutrition examination survey 2008. *Clin Exp Otorhinolaryngol* 2010; 3:183–193. 21217958 10.3342/ceo.2010.3.4.183 PMC3010536

[4] Knudsen LV Oberg M Nielsen C Factors influencing help seeking, hearing aid uptake, hearing aid use and satisfaction with hearing aids: a review of the literature. *Trends Amplif* 2010; 14:127–154. 21109549 10.1177/1084713810385712 PMC4111466

[5] Smeeth L Fletcher AE Ng ES Reduced hearing, ownership, and use of hearing aids in elderly people in the UK—the MRC Trial of the Assessment and Management of Older People in the Community: a cross-sectional survey. *Lancet* 2002; 359:1466–1470. 11988245 10.1016/s0140-6736(02)08433-7

[6] Smits C Kramer SE Houtgast T Speech reception thresholds in noise and self-reported hearing disability in a general adult population. *Ear Hear* 2006; 27:538–549. 16957503 10.1097/01.aud.0000233917.72551.cf

[7] Chien W Lin FR Prevalence of hearing aid use among older adults in the United States. *Arch Intern Med* 2012; 172:292–293. 22332170 10.1001/archinternmed.2011.1408 PMC3564585

[8] Kochkin S MarkeTrak VII: Obstacles to adult non-user adoption of hearing aids. *The Hear J* 2007; 60:24–50.

[9] Popelka MM Cruickshanks KJ Wiley TL Low prevalence of hearing aid use among older adults with hearing loss: the Epidemiology of Hearing Loss Study. *J Am Geriatr Soc* 1998; 46:1075–1078. 9736098 10.1111/j.1532-5415.1998.tb06643.x

[10] AmericanAcademy of Otolaryngology-Head and Neck Surgery Foundation I. Committee on Hearing and Equilibrium guidelines for the evaluation of results of treatment of conductive hearing loss. *Otolaryngol Head Neck Surg* 1995; 113:186–187. 7675477 10.1016/S0194-5998(95)70103-6

[11] Cruickshanks KJ Tweed TS Wiley TL The 5-year incidence and progression of hearing loss: the epidemiology of hearing loss study. *Arch Otolaryngol Head Neck Surg* 2003; 129:1041–1046. 14568784 10.1001/archotol.129.10.1041

[12] Ciorba A Bianchini C Pelucchi S The impact of hearing loss on the quality of life of elderly adults. *Clin Interv Aging* 2012; 7:159–163. 22791988 10.2147/CIA.S26059 PMC3393360

[13] Appollonio I Carabellese C Frattola L Effects of sensory aids on the quality of life and mortality of elderly people: a multivariate analysis. *Age Ageing* 1996; 25:89–96. 8670534 10.1093/ageing/25.2.89

[14] Mulrow CD Tuley MR Aguilar C Sustained benefits of hearing aids. *J Speech Hear Res* 1992; 35:1402–1405. 1494282 10.1044/jshr.3506.1402

[15] Turner CW Humes LE Bentler RA A review of past research on changes in hearing aid benefit over time. *Ear Hear* 1996; 17:14s–25s. 8807271 10.1097/00003446-199617031-00003

[16] Humes LE Wilson DL Barlow NN Changes in hearing-aid benefit following 1 or 2 years of hearing-aid use by older adults. *J Speech Lang Hear Res* 2002; 45:772–782. 12199406 10.1044/1092-4388(2002/062)

[17] Chao TK Chen TH Cost-effectiveness of hearing aids in the hearing-impaired elderly: a probabilistic approach. *Otol Neurotol* 2008; 29:776–783. 18725859 10.1097/MAO.0b013e31817e5d1b

[18] Gopinath B Schneider J Hartley D Incidence and predictors of hearing aid use and ownership among older adults with hearing loss. *Ann Epidemiol* 2011; 21:497–506. 21514179 10.1016/j.annepidem.2011.03.005

[19] Mizutari K Michikawa T Saito H Age-related hearing loss and the factors determining continued usage of hearing aids among elderly community-dwelling residents. *PLoS One* 2013; 8:e73622. 24086287 10.1371/journal.pone.0073622 PMC3781133

[20] van den Brink RH Wit HP Kempen GI Attitude and help-seeking for hearing impairment. *Br J Audiol* 1996; 30:313–324. 8922696 10.3109/03005369609076779

[21] Lim HW Chae SW The current state of age-related hearing loss in South Korea. *J Korean Med Assoc* 2011; 54:910–917.

[22] Hosford-Dunn H Halpern J Clinical application of the SADL scale in private practice II: predictive validity of fitting variables. Satisfaction with amplification in daily life. *J Am Acad Audiol* 2001; 12:15–36. 11214976

[23] Gussekloo J de Bont LE von Faber M Auditory rehabilitation of older people from the general population—the Leiden 85-plus study. *Br J Gen Pract* 2003; 53:536–540. 14694666 PMC1314644

[24] Tunkel DE Bauer CA Sun GH Clinical practice guideline: tinnitus. *Otolaryngol Head Neck Surg* 2014; 151:S1–S40. 25273878 10.1177/0194599814545325

[25] Del Bo L Ambrosetti U Hearing aids for the treatment of tinnitus. *Prog Brain Res* 2007; 166:341–345. 17956798 10.1016/S0079-6123(07)66032-4

[26] Moffat G Adjout K Gallego S Effects of hearing aid fitting on the perceptual characteristics of tinnitus. *Hear Res* 2009; 254:82–91. 19409969 10.1016/j.heares.2009.04.016

[27] Searchfield GD Kaur M Martin WH Hearing aids as an adjunct to counseling: tinnitus patients who choose amplification do better than those that don’t. *Int J Audiol* 2010; 49:574–579. 20500032 10.3109/14992021003777267

[28] Surr RK Montgomery AA Mueller HG Effect of amplification on tinnitus among new hearing aid users. *Ear Hear* 1985; 6:71–75. 3996787 10.1097/00003446-198503000-00002

[29] Lin FR Ferrucci L Hearing loss and falls among older adults in the United States. *Arch Intern Med* 2012; 172:369–371. 22371929 10.1001/archinternmed.2011.728 PMC3518403

[30] Rumalla K Karim AM Hullar TE The effect of hearing aids on postural stability. *Laryngoscope* 2015; 125:720–723. 25346316 10.1002/lary.24974

[31] Lach HW Incidence and risk factors for developing fear of falling in older adults. *Public Health Nurs* 2005; 22:45–52. 15670324 10.1111/j.0737-1209.2005.22107.x

[32] Gopinath B Rochtchina E Wang JJ Prevalence of age-related hearing loss in older adults: Blue Mountains Study. *Arch Intern Med* 2009; 169:415–416. 19237727 10.1001/archinternmed.2008.597

[33] Erber NP Use of hearing aids by older people: influence of non-auditory factors (vision, manual dexterity). *Int J Audiol* 2003; 42 Suppl 2:2s21–25. 12918625

[34] Meredith R Stephens D In-the-ear and behind-the-ear hearing aids in the elderly. *Scand Audiol* 1993; 22:211–216. 8146584 10.3109/01050399309047471

[35] Schneider J Dunsmore M McMahon CM Improving access to hearing services for people with low vision: piloting a “hearing screening and education model” of intervention. *Ear Hear* 2014; 35:e153–161. 24852681 10.1097/AUD.0000000000000038

[36] Simon HJ Levitt H Effect of dual sensory loss on auditory localization: implications for intervention. *Trends Amplif* 2007; 11:259–272. 18003869 10.1177/1084713807308209 PMC4111533

[37] Aazh H Prasher D Nanchahal K Hearing-aid use and its determinants in the UK National Health Service: a cross-sectional study at the Royal Surrey County Hospital. *Int J Audiol* 2015; 54:152–161. 25395258 10.3109/14992027.2014.967367

[38] Bainbridge KE Ramachandran V Hearing aid use among older U.S. adults; the national health and nutrition examination survey, 2005–2006 and 2009–2010. *Ear Hear* 2014; 35:289–294. 24521924 10.1097/01.aud.0000441036.40169.29 PMC3999213

[39] Dawes P Fortnum H Moore DR Hearing in middle age: a population snapshot of 40- to 69-year olds in the United Kingdom. *Ear Hear* 2014; 35:e44–51. 24518430 10.1097/AUD.0000000000000010 PMC4264521

[40] Hartley D Rochtchina E Newall P Use of hearing AIDS and assistive listening devices in an older Australian population. *J Am Acad Audiol* 2010; 21:642–653. 21376005 10.3766/jaaa.21.10.4

